# Influence of some parameters on the ability of *Listeria monocytogenes*, *Listeria innocua*, and *Escherichia coli* to form biofilms

**DOI:** 10.14202/vetworld.2019.459-465

**Published:** 2019-03-26

**Authors:** Sara Lezzoum-Atek, Leila Bouayad, Taha Mossadak Hamdi

**Affiliations:** 1 Laboratory of Food Hygiene and Quality Insurance System, High National Veterinary School, Algiers, Algeria; 2 Biology Department, Faculty of Sciences of Nature and Life and Earth Sciences, University of Bouira, Algeria

**Keywords:** biofilm, *Escherichia coli*, *Listeria innocua*, *Listeria monocytogenes*, polystyrene support, variation of parameters

## Abstract

**Aim:**

The present study was conducted to evaluate the capacity of *Listeria monocytogenes* (L.m), *Listeria innocua* (L.i), and *Escherichia coli* to form biofilms on polystyrene support under different parameters by performing crystal violet (CV) staining technique.

**Materials and Methods:**

Different suspensions were prepared with single strains and with multiple combinations of strains including two serogroups of L.m (*IIa* and *IIb*), L.i, and *E. coli* strains at different microbial load. Selected strains and combinations were grown in biofilms for 6 days attached to polystyrene microplates under aerobic and microaerophilic conditions. The evaluation of the power of adhesion and biofilm formation was determined by CV staining followed by the measurement of optical density at 24 h, 72 h, and 6 days incubation time with and without renewal of the culture medium.

**Results:**

All the strains tested, presented more or less adhesion power depending on the variation of the studied parameters as well as the ability to form multispecies biofilms. Their development is more important by renewing the culture medium and increasing the initial load of bacteria. The ability to adhere and form biofilms differs from one serogroup to another within the same species. In bacterial combination, strains and species of bacteria adopt different behaviors.

**Conclusion:**

The ability to form biofilms is a key factor in the persistence of tested strains in the environment. Our study showed that L.m, L.i, and *E. coli* could adhere to polystyrene and form biofilms under different conditions. More researches are necessary to understand the mechanisms of biofilm formation and the influence of different parameters in their development.

## Introduction

Biofilms existence and formation are well documented for many years. Any solid surface in contact with fluid containing bacteria is likely to be a biofilm carrier. Some authors estimate that biofilms represent the lifestyle of >99% of terrestrial bacteria [1]. Biofilms colonize various surfaces; they are particularly known for their adverse effects in the fields of public health and industry, their most known negative impacts are nosocomial diseases, contamination of food products, and biodeterioration of materials, particularly biocorrosion and biological fouling of industrial equipment [2]. A biofilm is defined as a community of microorganisms adhering to a surface and surrounded by a complex matrix of exopolysaccharides (EPSs) made of substances synthesized by these same microorganisms [3,4]. Its composition is carbohydrates, proteins, and other substances such as lipids or DNA [5]. The EPS provides protection to the microbial population of biofilms by concentrating nutrients, sequestering toxins, and preventing desiccation and biocides action [6]. The previous studies have confirmed the ability of different microbial species to form biofilms and persist in the environment, such as *Salmonella*, *Staphylococcus* [7,8], *Listeria monocytogenes* (L.m) [9], *Listeria innocua* (L.i) [10], *Escherichia* *coli* [11], and *Campylobacter* *jejuni* [12]. The ability of bacteria to form biofilms and their quantification was studied by applying different techniques including the use of polystyrene microplates [13] and crystal violet (CV) staining [14]. CV staining, originally described by Christensen *et al*. [15] and modified by Stepanovic *et al*. [16], allows the quantification of the biomass of a few hours or a few days old thin biofilms.

This study aimed to test the ability of L.m, L.i, and *E. coli* strains to form biofilms on 96-well polystyrene microplates by performing CV staining technique. Five influencing parameters were studied: Aerobic and microaerophilic conditions, microbial load, renewal of the culture medium, incubation time, and bacterial species composing the biofilm.

## Materials and Methods

### Ethical approval

Ethical approval was not needed for this study.

### Bacterial strains

The used strains of L.m and L.i were obtained from a previous study [17]. Six serogrouped and pulsotype strains of L.m were tested, among them, three strains (L.m 026, L.m 038, and L.m 055) belonged to the same serogroup *IIa* (L.m/*IIa*) and the same pulsotype; two strains (L.m 023 and L.m 043) to serogroup *IIa*, but each one to a different pulsotype and one strain (L.m 036) belonged to serogroup *IIb* (L.m/*IIb*) and had its pulsotype. *E. coli* strains were collected from surfaces in a slaughterhouse according to the ISO 18593 (2004) method [18]. Isolation and identification of these strains were carried out on chromID™ Coli agar (COLI ID-F) (bioMérieux) according to the recommendations of the ISO 4832 (2006) method [19]. The putative colonies were confirmed using the API 20E strip.

### Culture media and growing conditions

The adhesion power and the ability of bacteria to form biofilm were carried out in two stages. The first one was to prepare different bacterial suspensions from different species and different strains, and the second one is to test the capacity of adhesion and biofilm formation of the different bacterial suspensions on polystyrene microplates by performing a CV staining (stain which binds to bacteria and EPS in the extracellular matrix of the biofilm) [20]. The bacteria were revivified by streaking onto Trypticase Soy Agar (bioMérieux) for 24 h at 37°C and enriched at 37°C for 24 h on Trypticase Soy Broth (TSB) (bioMérieux) to produce bacterial suspensions. The influence of the bacterial load, strains and species combinations and their concentrations on the ability of bacteria to adhere and form biofilms (single strain, single species, and multispecies) were investigated. On this purpose, bacterial suspensions were prepared with concentration adjusted to 1 and 4 McFarland using a densitometer (DensiCHEK Plus Instrument, bioMérieux). The 1 McFarland bacterial suspensions were prepared from the single strains and from the following combinations: L.m/*IIa*, L.m/*IIa*+*L.i*, L.m/*IIb*+*L.i*, L.m/*IIa*+ *E. coli*, L.m/*IIb*+*E*. *coli*, L.m./*IIa*+*L.i*+*E*. *coli*, L.m/*IIb*+ *L.i*.+*E*. *coli*, and *L.i*+*E*. *coli*. The suspensions at 4 McFarland were prepared from strains alone and from the following combinations: L.m/*IIa*+*E*. *coli*, L.m/*IIb*+*E*. *coli*, and *L.i*+*E*. *coli*. The obtained suspensions were seeded for each experimentation on eight triplicate polystyrene microplates. Measurements of the optical density (OD) at 550 nm were realized at 24 h to estimate the adhesion power. Two further readings were performed after 72 h and 6 days of incubation to estimate the ability of strains to form biofilms [ 21-23]. The method of assessing biofilm formation was based on the technique described by Stepanović *et al*. [16,24] and adapted for the analysis of selected strains and combinations. 200 µl of each suspension were inoculated into triplicates; the last three columns were filled with 200 µl of TSB to serve as controls. To test the effect of the oxygen content on the adhesion and the biofilms formation, four microplates were incubated in aerobic conditions while others in microaerophilic conditions using GENbox microaer and GENbag microaer (bioMérieux) for 6 days at 37°C. The first reading of the ODs was performed after 24 h of incubation. One aerobic incubated plate and one microaerophilic incubated plate were retained to achieve a renewal of the culture medium to evaluate its effect on the development of biofilms. The wells were gently overturned and then filled with 200 µl of TSB. The retained plates for this test were subsequently incubated with the other plates for 96 h. Before measuring the OD, the wells were aspirated, rinsed with sterile physiological water 3 times, and fixed by adding 200 µl of methanol to each well. After 15 min, the plates were overturned, dried at ambient temperature, and then stained by adding 200 μl of 2% CV solution (bioMérieux). After 5 min, the plates were rinsed with running water 5 times and dried at room temperature. The OD corresponding to each bacterial species, strains, and combination of species (OD_s_) was obtained by calculating the average of the triplicates, then compared with the averages of the ODs of the control wells (ODcn) [25]. The evaluation of the power of adhesion and biofilm formation is performed by applying the following classification: No biofilm production if ODs≤ODnc; low biofilm production when ODnc <ODs ≤2. ODnc; moderate (M) production of biofilm 2.ODnc <ODs ≤ 4. ODnc and strong (S) biofilm production 4.ODnc <ODs [13,26,27].

### Statistical analysis

The XLSTAT-Premium software was used to analyze the OD values collected by applying a one-way analysis of variance. The averages were considered significantly different for values of p<0.05.

## Results

### Formation of single-species biofilms

In aerobic conditions and at 1 McFarland concentration, all strains of L.m and L.i showed a strong adhesion after 24 h of incubation and a biofilm formation at 72 h, except the strain L.m 038 (*IIa*) which showed a medium adhesion after 24 h, then a strong biofilm production after 72h and for the strain L.m 036 (*IIb*) which showed a medium adhesion after 24 h and a medium production of biofilm at 72 h and after 6 days (Table-1).

**Table-1:** Results of adhesion and biofilm formation tests of L.m, L.i, and *E. coli* strain at 1 McFarland suspensions.

Strains	L.m									L.i			*E. coli*		

	023,026, 043,055			038			036								
			
Serogroup	*IIa*			*IIa*			*IIb*								
Incubation time	24 h	72 h	6 d	24h	72 h	6 d	24 h	72 h	6 d	24 h	72 h	6 d	24 h	72 h	6 d
Aerobic conditions	S	S	S	M	S	S	M	M	M	M	S	S	S	S	S
Microaerophilic conditions	M	S	S	M	S	S	S	S	S	M	S	S	S	S	S

S=Strong, M=Moderate, h=Hours, d=Days, *E. coli*=*Escherichia coli*, L.m=*Listeria monocytogenes*, L.i=*Listeria innocua*

**Figure-1:**
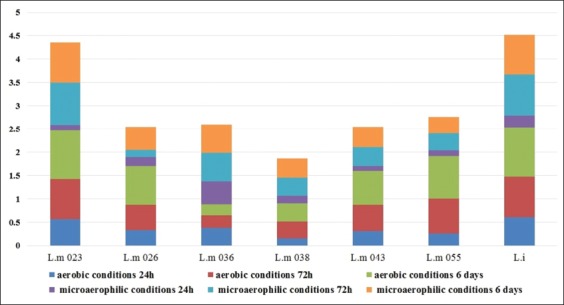
Effect of aerobic and microaerophilic conditions on biofilm formation from 1 McFarland suspensions. Captions: L.m: *Listeria monocytogenes*, L.i: *Listeria innocua*, OD: Optical density, h: Hour.

In microaerophilic conditions, all strains of L.m and L.i tested showed a medium adhesion after 24 h of incubation, followed by a strong biofilm production after 72 h, except for the strain L.m 036 (*IIb*) which already showed a strong adhesion from the first 24 h. Adhesion and biofilm formation of *E. coli* in aerobic or microaerophilic conditions were strong. However, biofilms development was more important in microaerophilic than in aerobic conditions (p<0.05) (Figure-1).

At 4 McFarland concentration, all the strains of L.m, L.i, and *E. coli* showed a strong adhesion from the first 24 h of incubation and biofilms formation, in aerobic or microaerophilic conditions. The development of *E. coli* strains was higher in microaerophilic than in aerobic conditions (p<0.05).

### Formation of multistrain and multispecies biofilms

In aerobic conditions, all stains combination tested at 1 McFarland showed a strong adhesion from the first 24 h of incubation with a large formation of biofilm. In microaerophilic conditions, the adhesion was strong at 24 h for all the strain combination except for the combination of L.m 038+L.m 026+L.m 043 (L.m/*IIa*) which showed a moderate adhesion after 24 h of incubation and for the combination of L.m 023+L.m 026+Li (L.m/*IIa*+L.i) which showed a moderate adhesion and M biofilm production after 72 h of incubation. All combinations of bacterial strains tested at 4 McFarland showed a strong adhesion from the first 24 h of incubation with a large biofilm formation in aerobic and microaerophilic conditions.

### Effect of the renewal of the culture medium

Renewal of the culture medium on 1 McFarland suspensions has given a higher development of biofilms in both aerobic and microaerophilic conditions. OD measurements at 72 h of incubation with the renewal of the culture medium gave similar results to those obtained with the same strains after 6 days of incubation without renewal of the culture medium.

### Effect of incubation time

**Figure-2:**
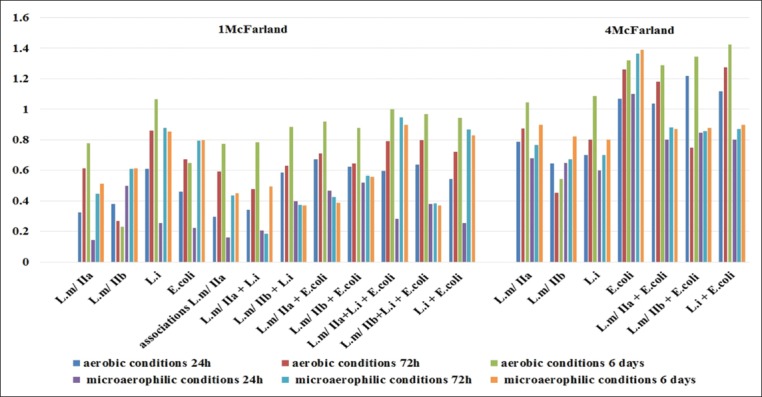
Effect of the incubation time on biofilms formation. Captions: L.m: *Listeria monocytogenes*, L.i: *Listeria innocua*, OD: Optical density, h: Hour.

Measurements of OD at 24 h, 72 h, and 6 days showed that the development of biofilms increased significantly during the first 72 h. This increase tends to stabilize during the followed 72 h. The results of the OD measurements after 6 days of incubation showed a significant difference compared to those obtained after 72 h of incubation for most strains (p<0.05) (Figure-2).

## Discussion

### Formation of single-species biofilms

The previous studies have reported that species of the genus *Listeria* and the genus *E*. *coli* can form biofilms on different types of surfaces [9,26,28]. Our study showed that strains of L.m and L.i could adhere to polystyrene and form biofilms under aerobic and microaerophilic conditions. *Listeria* species at 1 McFarland concentration showed that the microaerophilic conditions slow down the adhesion of most strains during the first 24 h; however, the slowdown is followed by a relatively large development of biofilms throughout the incubation period. This behavior can be explained by the need for an adaptation time for strains to microaerophilic conditions. At the end of the 6 days incubation, there was no significant difference (p>0.05) between the development of biofilms, whether in aerobic or microaerophilic conditions. This is probably due to the ability of *Listeria* to develop in atmospheres with slightly lower oxygen tension and increased carbon dioxide than air [29,30]. All L.m strains belonging to the serogroup *IIa* (Table-1) showed almost the same adhesion power, which led to biofilm formation after 72 h of incubation, except L.m 038 strain which showed at the beginning (24 h) of moderate adhesion. This suggests that within the same serogroup, the time spent to adapt to microaerophilic condition and biofilm formation is different.

L.m 036 strain, which belongs to the serogroup *IIb*, exhibited different behavior, developing more in microaerophilic than aerobic conditions, with less adhesion and medium biofilm formation (p<0.05) compared to other strains. Our results corroborate those obtained by Borucki *et al*. [31] and Pan [32], who found that L.m strains belonging to serogroup *IIa* had a higher ability to form biofilms. The study results of Kadam *et al*. [28] and Kalmokoff *et al*. [33] also showed that under certain conditions, the ability of L.m to adhere to surfaces could be related to the serogroup factor.

L.i strains showed the same adhesion power as L.m. These characteristics have been reported by Kalmokoff *et al*. [33], Robitaille *et al*. [34], and Jeon *et al*. [10]. They have reported the ability of L.i to adhere to several types of surfaces and their high adhesion power is similar to that of L.m strains.

The results also showed that *E*. *coli* strains could adhere to polystyrene and form biofilms under aerobic and microaerophilic conditions. Development was statistically better under microaerophilic conditions (p<0.05). These results corroborate those of the previous studies that reported the ability of *E. coli* to form biofilms on polystyrene under different conditions [35]. However, this result differs from that reported by Auger [26], who had observed that anaerobic conditions slowed the development of biofilms considerably. This difference could be related to the fact that our tests were performed in microaerophilic and not in anaerobic conditions.

All the suspensions at 4 McFarland concentration showed a strong and early adhesion. This could be due to the high number of bacterial cells in contact with the surface, which has favored the adhesion and formation of biofilms. Microaerophilic conditions had allowed better adhesion and formation of biofilms for *E. coli* strains. This could be due to the ability of these strains to adapt to these conditions.

### Formation of multispecies biofilms

Combinations of strains tested at both 1 and 4 McFarland concentrations, allowed the formation of strong biofilms whether in aerobic or microaerophilic conditions. This could be explained by the ability of strains to coexist in a multispecies biofilm and a probable synergy between them. In the previous studies, Jeong and Frank [36] and Chmielewski and Frank [6] reported the ability of certain pathogens to coexist within a single biofilm. The ability of L.m to grow within a multispecies biofilm has been reported by Chen *et al*. [37] who worked on biofilms formed by *Salmonella typhimurium*, STEC, and L.m and reported by Buchanan *et al*. [38]. The aptitude of *E. coli* to form multispecies biofilms has been described since 2008 [39]. Numerous examples of synergistic induction of multispecies biofilm formation constituted by *E. coli* and other species were reported by many studies such as Romeo [39], Giaouris *et al*. [40] and [41], and Larsen *et al*. [42]. They described the synergistic effect between different bacterial strains composing a multispecies biofilm among them L.m and *E. coli*. The impact of interspecies communication on biofilm development is presently not well understood [39].

Our study showed some particularities. The combination of L.m 038+ L.m 026 + L.m 043 (L.m/*IIa*) showed a moderate adhesion after 24 h of incubation. This slow adhesion could be linked to the presence of L.m 038 strain which has already shown a moderate adhesion at 24 h when it has been tested alone (biofilm monospecies). The combination of L.m 023+L.m 026+L.i (L.m/*IIa*+L.i) has shown a moderate adhesion after 72 h of incubation, knowing that when tested alone, each strain showed a strong adhesion at 24 h. This suggests that bacterial strains may show different behavior when they are simultaneously present in a multispecies biofilm, either by increasing or decreasing the adhesion capacity. This difference in behavior of L.m was reported by Giaouris *et al*. [41] and Jay *et al*. [43].

### Effect of the renewal of the culture medium

The renewal of the culture medium has led to a higher biofilms development in aerobic and microaerophilic conditions. These results corroborate those obtained by Auger [26], who had noted that glucose supplementation promotes the development of biofilms. Due to its composition, TSB allowed an additional nutrient supply which likely improved the bacterial growth conditions. These results are similar to that obtained by Stepanović *et al*. [16], who found that L.m forms stronger biofilms in the presence of a nutrient-rich environment. Zeraik and Nitschke [44] and Azam *et al*. [35] showed that supplementation of culture medium with various substances influenced significantly their ability to form biofilms.

### Effect of incubation time

Incubation time is an important factor in the process of biofilm formation [45]. The adhesion of the strains tested was observed after 24 h of incubation. The formation of biofilms was more important during the first 72 h of incubation to give a biofilm that tended to stabilize during the past 72 h. Jay *et al*. [43] reported that biofilms of L.m could reach their maximum development after 72 h of incubation in optimum conditions. Our results are similar to those obtained by Han *et al*. [23], who showed that the mass of *E. coli* formed biofilms on the polystyrene surface increases with the incubation time. The previous studies of L.m biofilms have shown that after rapid initial adhesion to surfaces, bacterial populations do not increase significantly [9].

## Conclusion

All the strains tested showed a more or less adhesion power depending on the variation of the studied parameters as well as an ability to form multispecies biofilms. Strains of L.m, L.i, and *E. coli* adhere to polystyrene and form biofilms under aerobic and microaerophilic conditions. These biofilms develop well during the first 72 h and are influenced by microaerophilic incubation, especially those of *E. coli*. Their development is even more important by renewing the culture medium and increasing the initial load of bacteria (4 McFarland).

The ability to adhere and form biofilms is different from one serogroup to another within the same species. In bacterial combination, strains and species adopt different behaviors. The ability of bacterial species to form biofilms poses a real problem in different areas. The study of the ability of strains to adhere to surfaces and to form biofilms remains complex. Several parameters can influence the adhesion, such as the nature of the surfaces, the bacterial species, the number of bacteria, the presence of a single bacterial genus or multi-genus, and many other parameters.

Recent advances in the understanding of the biofilm development cycle have indicated that, in most cases, it is a dynamic process in which factors such as nutritional conditions, temperature, oxygen tension, and osmolarity can have big influences on biofilm formation. The variation of these parameters can influence the adhesion power of bacteria as well as the extent of biofilm formation.

More researches are still necessary to understand the mechanisms of biofilm formation and the influence of different parameters in their development. The next stage of our research will involve testing the effect of some biocides on the viability of species forming biofilms and their sensitivity to antibiotics with the aim of studying the probable correlation between sensitivity toward biocides and antibiotics.

## Authors’ Contributions

SLA planned the study and drafted the manuscript under the supervision of LB and TMH. SLA designed the experiment protocol under the supervision of LB and TMH. SLA collected and analyzed samples did the statistical analysis and revised the manuscript under the supervision of LB and TMH. All authors read and approved the final manuscript.
